# Transfer Learning Video Classification of Preserved, Mid-Range, and Reduced Left Ventricular Ejection Fraction in Echocardiography

**DOI:** 10.3390/diagnostics14131439

**Published:** 2024-07-05

**Authors:** Pierre Decoodt, Daniel Sierra-Sosa, Laura Anghel, Giovanni Cuminetti, Eva De Keyzer, Marielle Morissens

**Affiliations:** 1 Cardiologie, Centre Hospitalier Universitaire Brugmann, Faculté de Médecine, Université Libre de Bruxelles, Place Van Gehuchten 4, 1020 Brussels, Belgium; lauraionela.anghel@chu-brugmann.be (L.A.); giovanni.cuminetti@chu-brugmann.be (G.C.); evalisa.dekeyzer@chu-brugmann.be (E.D.K.); marielle.morissens@chu-brugmann.be (M.M.); 2 Computer Science and Information Technologies Department, Hood College, 401 Rosemont Ave., Frederick, MD 21702, USA; sierra-sosa@hood.edu

**Keywords:** medical imaging, echocardiography, diagnosis, cardiovascular diseases, heart failure, cardiac function, left ventricular ejection fraction, machine learning, transfer learning, AutoML

## Abstract

Identifying patients with left ventricular ejection fraction (EF), either reduced [EF < 40% (rEF)], mid-range [EF 40–50% (mEF)], or preserved [EF > 50% (pEF)], is considered of primary clinical importance. An end-to-end video classification using AutoML in Google Vertex AI was applied to echocardiographic recordings. Datasets balanced by majority undersampling, each corresponding to one out of three possible classifications, were obtained from the Standford EchoNet-Dynamic repository. A train–test split of 75/25 was applied. A binary video classification of rEF vs. not rEF demonstrated good performance (test dataset: ROC AUC score 0.939, accuracy 0.863, sensitivity 0.894, specificity 0.831, positive predicting value 0.842). A second binary classification of not pEF vs. pEF was slightly less performing (test dataset: ROC AUC score 0.917, accuracy 0.829, sensitivity 0.761, specificity 0.891, positive predicting value 0.888). A ternary classification was also explored, and lower performance was observed, mainly for the mEF class. A non-AutoML PyTorch implementation in open access confirmed the feasibility of our approach. With this proof of concept, end-to-end video classification based on transfer learning to categorize EF merits consideration for further evaluation in prospective clinical studies.

## 1. Introduction

Worldwide, heart failure is one of the most concerning cardiac conditions, characterized by impaired quality of life, serious complications, and a high mortality rate [1]. However, progress is constantly observed in therapy, especially since the development of dedicated units ensuring patient follow-up, and numerous observations show that the prognosis can be improved by adapting the therapeutic measures to the impairment of left ventricular function [2,3,4]. The left ventricular ejection fraction (EF) is defined as the percentage of blood present at end-diastole in the left ventricle that is ejected during systole. It is the most evaluated parameter for classifying patients. The distinction was historically first made between patients with preserved EF (pEF,) where EF is not less than 50%, and not preserved EF (npEF). More recently, a distinction has been made in npEF patients between those with reduced EF (rEF), where the EF is less than 40%, and those with mid-range EF (mEF), where EF is between 40% and 50% [5,6]. Probably more appropriately, mid-range EF is sometimes referred to as mildly reduced EF.

Transthoracic echocardiography, a non-invasive, easily portable, and irradiation-free method, is the most widely used means of assessing EF. However, the method has some limitations compared to other heart-imaging techniques, like contrast ventriculography, computed tomography, magnetic resonance imaging, or positron emission tomography. Obtaining a good estimate of EF requires carrying out a delineation of the left ventricular cavity in end-diastole and end-systole on several consecutive beats, followed by volume estimation using the Simpson method of slice. This is a lengthy process that requires considerable human expertise.

Therefore, many machine-learning (ML) algorithms for EF measurement have been developed [7]. These models have mainly been tested on two publicly available labeled datasets, EchoNet-Dynamic [8] and CAMUS [9]. Most of these approaches require an initial human and/or ML intervention to determine the contours of the left ventricular cavity. The expertise of the practitioner or the AI performance is essential in this process.

The receiver operating characteristic curve area under the curve (ROC AUC) score in a binary npEF vs. pEF classification is reported in two studies. Using the EchoNet-Dynamic three-component algorithm, the ROC AUC score was 0.97 for the institutional Stanford dataset and 0.96 for an external dataset obtained at Cedars-Sinai Medical Center [10]. For the same classification task, ROC AUC scores of around 0.97 were obtained from datasets derived from the CAMUS repository using DPS-Net, a segmentation algorithm, in conjunction with the Simpson biplane method [11]. Ten-fold cross-validation was performed in the latter study. More recently, transfer learning was proposed to increase the performance of the part of algorithms allowing performing the segmentation [12,13].

A different approach not yet described to the best of our knowledge consists of submitting the echocardiographic video sequences showing the beating heart without any phase of segmentation to a transfer learning algorithm of video classification. These algorithms are presently accessible either in free open-access packages or in the torchvision.models subpackage of PyTorch to run on personal or institutional computer systems or through pay access directly to the cloud via automated machine learning (AutoML) or customized code in Google Vertex AI.

Our objective was to evaluate whether an end-to-end transfer learning video classification, without human or AI determination of the left ventricular location or internal contours, is feasible and performs well in distinguishing patients with pEF, mEF, and rEF. This approach presents a certain analogy with a classification of celestial objects based on the amplitude of their pulsation, hence its name, “PulseHeart”. The source of echocardiograms was the EchoNet-Dynamic dataset, which consisted of video sequences labeled by experts using the Simpson method. We created three balanced echocardiographic video datasets of different sizes (small: S-set, medium: M-set, large: L-set). Each set was subjected to three AutoML models with either binary (respectively, rEF vs. nrEF and npEF vs. pEF) or ternary (pEF, mEF, rEF) classification. Finally, we submitted the test video labels of the S-set to human re-evaluation.

## 2. Materials and Methods

**Figure 1:**
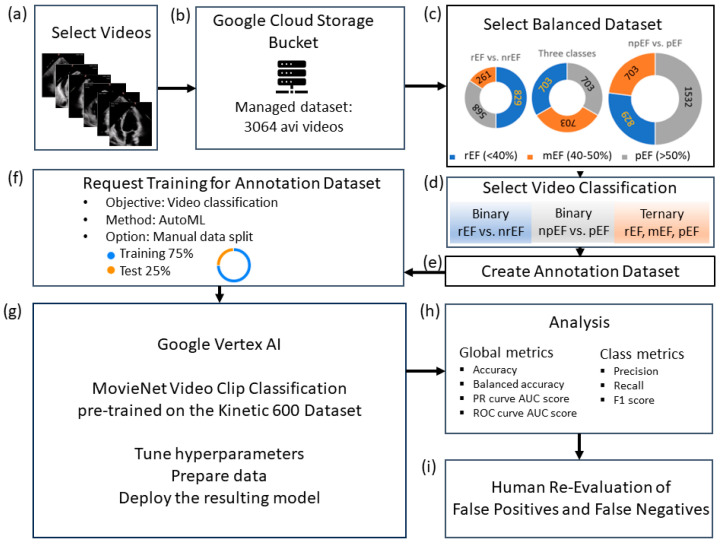
Study flowchart: (**a**) data curation; (**b**) upload the study-managed dataset to Google Cloud Storage; (**c**) balanced data sets S, M, and L, the size of which depends on the minority class (rEF, mEF, and npEF, respectively); (**d**): video classifications to test; (**e**): create a per-model annotation dataset and upload the corresponding CSV files (one for the train and one for the test videos); (**f**) specify the model to be trained; (**g**) run the model for training; (**h**) analyze the test set; (**i**) human panel reassessment of false positives and false negatives.

The high-level design is shown in Figure 1.

### 2.1. Dataset and Data Curation

**Figure 2:**
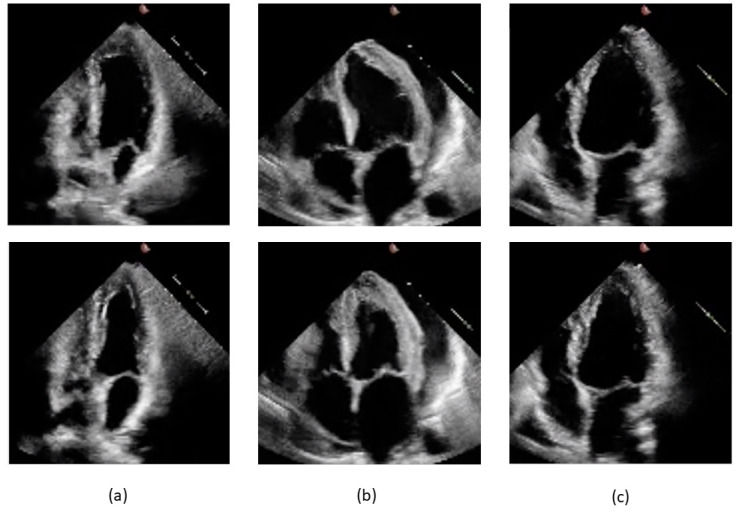
Frames from videos pertaining to the study dataset. End-diastolic frames are shown on the upper part of the slide and end-systolic frames below: (**a**) 0X2753C50A8B05D7D5.avi, label pEF, EF 58.3% by Simpson method; (**b**) 0X2F3141F00A232601.avi, label mEF, EF 42.1% by Simpson method; (**c**) 0X41563E2CC2230C0E.avi, label rEF, EF 21.9% by Simpson method.

The datasets were obtained from the EchoNet-Dynamic database, comprising 10,030 echocardiographic video sequences preprocessed, deidentified, and converted from DICOM to AVI format. Examples of end-diastolic and end-systolic four-chamber views for cases labeled pEF, mEF, and rEF are shown in Figure 2.

**Figure 3:**
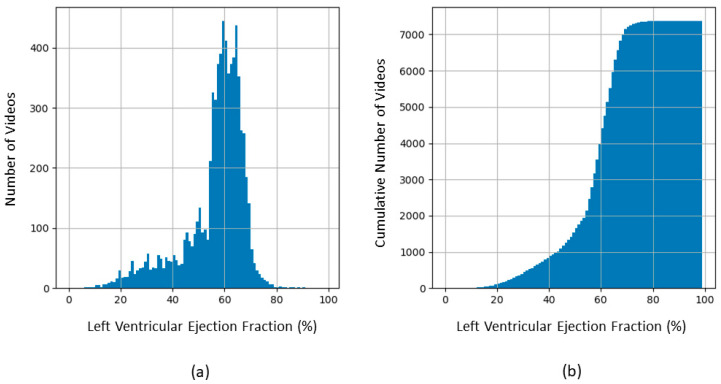
Distribution of EF in the 7380 videos of the intermediate dataset before balancing to create the S, M, and L sets. (**a**) Frequency histogram; (**b**) Cumulative histogram.

In the first curation step, we eliminated the 40 videos that were submitted by the Stanford group for re-evaluation by expert clinicians because of the highest absolute difference between the initial human label and the EchoNet-Dynamic’s prediction. In the second step, we restricted the set to the 112 × 112-pixel videos at 50 frames per sec with 100 to 250-frame lengths to obtain an intermediate dataset. Figure 3 shows the distribution of the EF values of this intermediate dataset of 7380 videos.

In the third step, the S-set, M-set, and L-set were obtained by majority undersampling as described in Table 1. The size of each dataset is determined by the maximal size of the considered minority (rEF, mEF, and npEF, respectively).

Unlike the EchoNet-Dynamic algorithm, the video classification by AutoML does not include a validation set; therefore, we grouped the video originally tagged VAL and TEST in the EchoNet-Dynamic database into the test category to obtain a 75/25 train–test split. The study’s 3064 videos were uploaded in a Google Cloud Storage bucket folder.

### 2.2. Training

The training was performed using the MoViNet Video Clip Classification (tfvision/movinet-vcn, release date: 20 August 2023), a task-specific solution implemented in Google Vertex AI and pre-trained on the Kinetic 600 dataset. Using Vertex AI training pipelines, we created managed datasets and corresponding AutoML-trained models for video classification (Table 2).

### 2.3. Analysis

For performance, we used the following metrics: accuracy, balanced accuracy, PRC AUC score, ROC AUC scores, and, for each class, precision, recall, and F1 score. The confusion matrix graphs were obtained by importing the results in a Python 3 Jupyter notebook with the help of Matplotlib’s pyplot module and Seaborn’s heatmap module. The Vertex AI output includes graphs of the precision-recall curve (PRC) and precision-recall by threshold curves (PRTC). These will be presented here. By reviewing the videos and noting each prediction, we were able to obtain graphs of ROC curves and calculate the corresponding AUC scores. For this purpose, we used the roc_curve and roc_auc_score modules of scikit-learn in Python.

### 2.4. Clinician Re-Evaluation

This re-evaluation was carried out for the three S-set experiments by a panel of five cardiologists who specialize in cardiovascular imaging (E.D., G.C., L.A., M.M., P.D.). A video subset was formed by grouping all false positives and false negatives observed in the two binary classifications and by adding the rEF cases predicted as pEF and the pEF cases predicted as rEF by the ternary classification. These videos were presented in random order to the panel members, who blindly classified them as rEF, mEF, and pEF. The majority rule was used for ranking, with mEF chosen in the event of a tie. Determination of poor technical quality and the presence of arrhythmia were also based on the majority rule. To explore the role of technical quality and arrhythmia at recording time, we calculated for each case the number of rater reports of degraded quality (Q-score, from 0 to 5) and of suspicion of arrhythmia (A-score, from 0 to 5). We used a two-tailed Student’s *t*-test to assess if these scores were different in underestimation vs. overestimation on EF by at least one of our classifiers.

## 3. Results

### 3.1. Binary Classification

The metric values for these six models are shown in Table 3. In general, the performance seems better with the S-set. Since a “positive” medical test means belonging to the class indicating disease, sensitivity is the recall for rEF in the rEF vs. nrEF classification and the recall for npEF in the npEF vs. pEF classification. Likewise, specificity is the recall for nrEF in the rEF vs. nrEF classification and the recall for pEF in the npEF vs. pEF classification.

#### 3.1.1. rEF vs. nrEF

**Figure 4:**
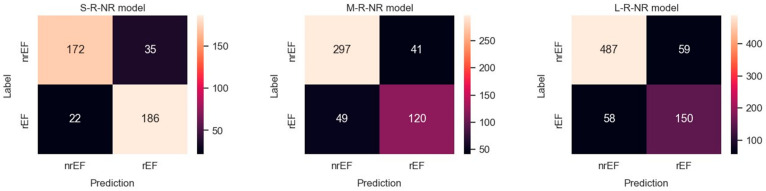
Confusion matrices observed for predicting rEF vs. nrEF using three differently balanced datasets.

**Figure 5:**
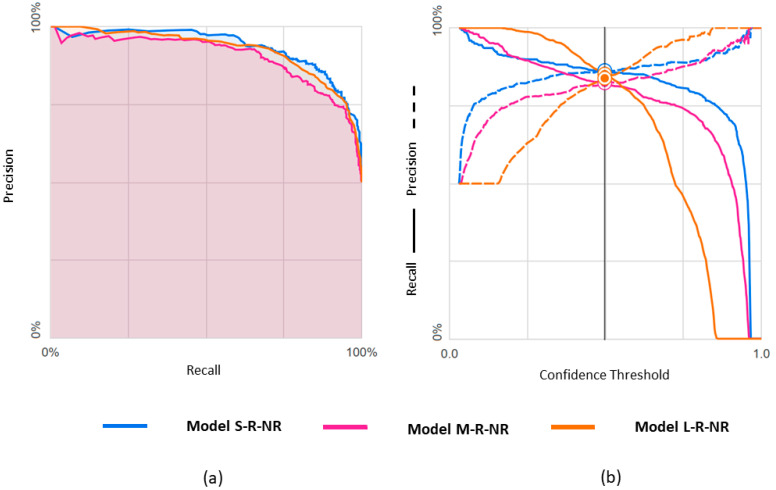
Predicting rEF vs. nrEF using three differently balanced datasets: (**a**) Precision-recall curve; (**b**) Precision-recall by threshold.

Results obtained with the three datasets are shown in terms of confusion matrices in Figure 4 and in terms of PRC and PRTC in Figure 5. Please note that the rEF class is the minority in these models. The S-set obtained by undersampling the mEF and pEF classes is the appropriate, balanced option for this binary classification (S-R-NR model).

#### 3.1.2. npEF vs. pEF

**Figure 6:**
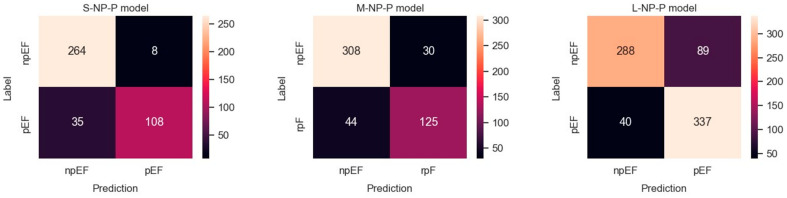
Confusion matrices observed for predicting npEF vs. pEF using three differently balanced datasets.

**Figure 7:**
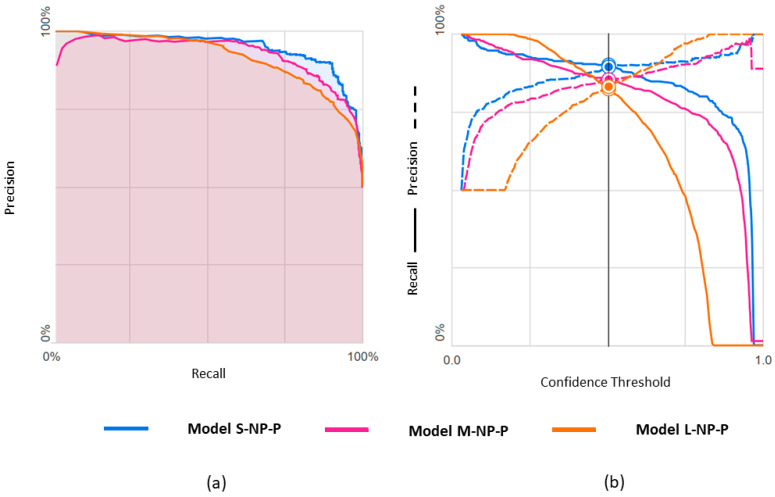
Predicting npEF vs. pEF using three differently balanced datasets: (**a**) Precision-recall curve; (**b**) Precision-recall by threshold.

Results obtained with the three datasets are shown in terms of confusion matrices in Figure 6 and in terms of PRC and PRTC in Figure 7. Please note that the npEF class is the minority in these models. The L-set obtained by undersampling the pEF class is the appropriate, balanced option for this binary classification (L-NP-P model).

#### 3.1.3. ROC Curves for Models with Binary Classification

**Figure 8:**
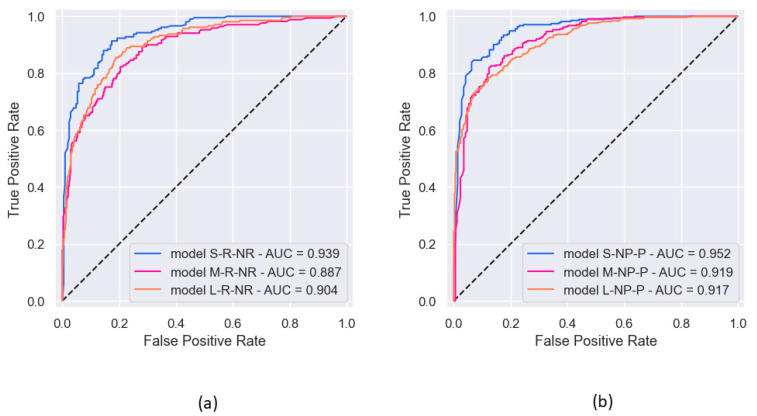
ROC curves observed in the six experiments of binary classification, along with the corresponding AUC: (**a**) prediction of rEF vs. nrEF; (**b**) prediction of npEF vs. pEF.

Figure 8 shows the ROC curves for the six models with binary classification. Pearson’s correlation was 0.909 between ROC AUC scores and PRC AUC scores.

### 3.2. Ternary Classification

**Figure 9:**
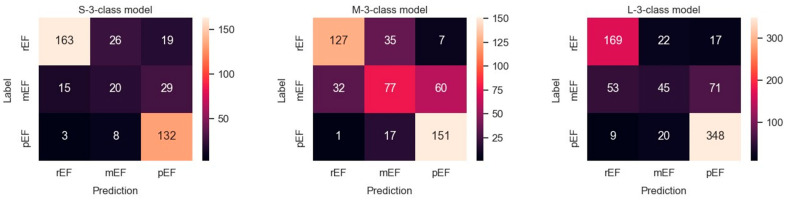
Confusion matrices observed in a ternary classification of the classes rEF, mEF, and pEF using three differently balanced datasets.

**Figure 10:**
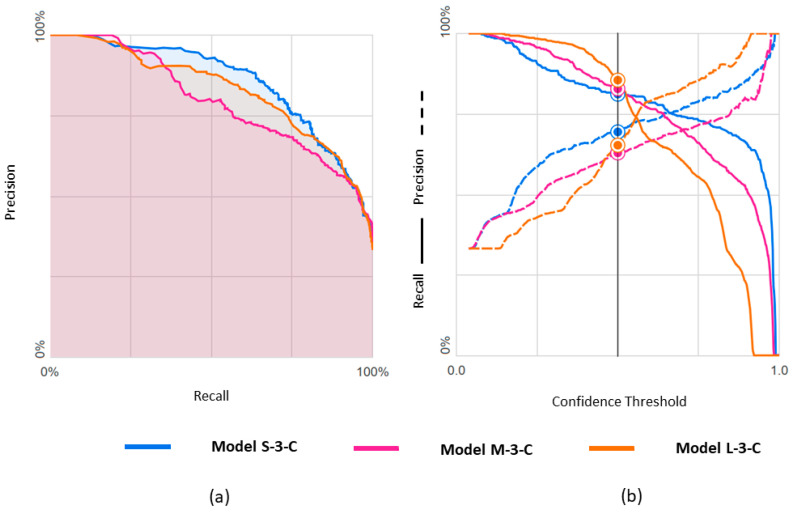
Predicting rEF, mEF, and pEF using three differently balanced datasets: (**a**) Precision-recall curve; (**b**) Precision-recall by threshold.

The metric values for these three models are shown in Table 4. Results obtained with the three datasets are shown in terms of confusion matrices in Figure 9 and in terms of PRC and PRTC in Figure 10. Please note that the mEF class is the minority in these models. The M-set obtained by undersampling the rEF and pEF classes is the appropriate option for this ternary classification (M-3-C model).

### 3.3. Clinician Re-Evaluation

Table A1 in Appendix A displays the detailed results of the re-evaluation. The recording quality was found to be poor in 29.9% (32 of 107) of these cases of mismatch between label and prediction, and arrhythmia was judged present in 7.5% (8 of 107). For comparison, in the clinician re-evaluation performed on the EchoNet-Dynamic’s prediction model [10], the experts rated 32.5% (13 of 40) of videos as having poor image quality and 13% (5 of 40) of videos as showing arrhythmia.

**Figure 11:**
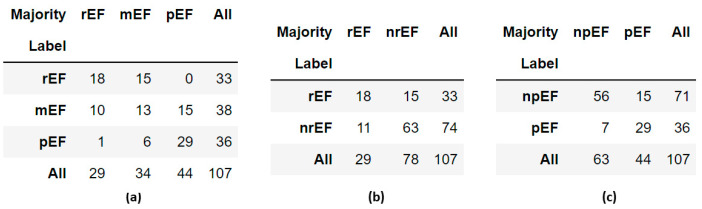
Cross-tables comparing labels obtained by the Simpson method with panel majority in the 107 reviewed videos: (**a**) ternary classification; (**b**) binary classification, rEF vs. nrEF; (**c**) binary classification, npEF vs. pEF.

The cross-tables (Figure 11) indicate that the panel judged that the EF was preserved in more cases than established by the original label and that the EF was reduced in fewer cases. However, this is mostly concerned with cases labeled mEF. A video labeled pEF was classified as rEF by three members of the panel, mEF by the fourth, and pEF by the last. No videos labeled rEF were classified as pEF by the majority. Such a paucity of possible “extreme” labeling errors (rEF instead of pEF or vice versa) was also observed when re-evaluating the EchoNet-Dynamic prediction model [10]. The discordance table published in that article shows that only 2 out of 40 human rEF labels should be replaced by pEF, and only three pEF labels should be replaced by rEF.

**Figure 12:**
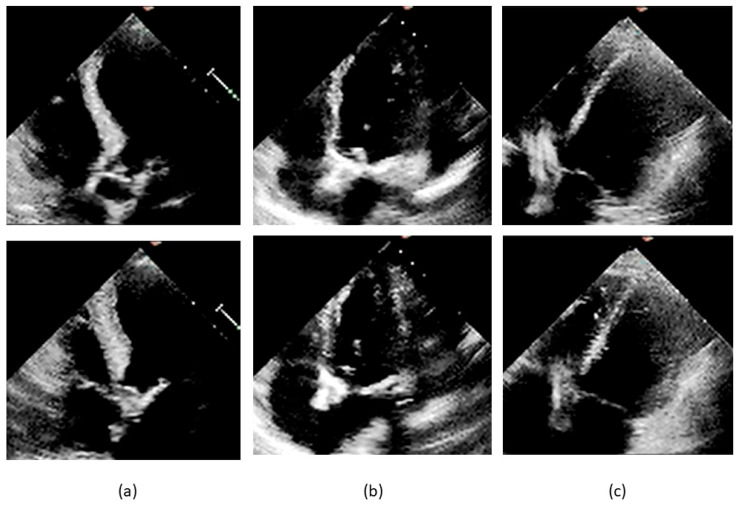
Frames from misclassified videos. End-diastolic frames are shown on the upper part of the slide and end-systolic frames below: (**a**) 0X41ECEC7AAEEFD0E6.avi, label pEF, EF 57.3% by Simpson method, pEF by unanimous panel, Q-score 5, A-score 0, misclassified as rEF; (**b**) 0X7923B6B4614AF456.avi, label mEF, EF 49.1% by Simpson method, mEF for four raters and rEF for one rater, Q-score 3, A-score 0, misclassified as rEF; (**c**) 0X3503A92D7637451.avi, label rEF, EF 31.8% by Simpson method, rEF by unanimous panel, Q-score 3, A-score 0, misclassified as nrEF.

The Q-score was not significantly different in the subgroups of EF overestimation (*n* = 43) and underestimation (*n* = 63) by classifier (means ± SD: 1.88 ± 1.69 vs.1.65 ± 1.41, 104 df, t = 0.767, *p* = 0.444). The A-score was significantly higher in the overestimation subgroup (means ± SD: 0.70 ± 1.28 vs. 0.27 ± 0.79, 104 df, t = 2.128, *p* = 0.036). Figure 12 presents three misclassified cases reported of poor quality by at least three members of our re-evaluation panel. The original labels were, respectively, pEF, mEF, and rEF. The comparison with correctly classified cases of good quality, as those presented in Figure 2, allows a quantitative insight into the variation of the visualization of the left ventricular internal contour across the dataset.

### 3.4. GitHub PyTorch Implementation

We conducted an additional series of experiments using open-access software, in which we were able to optimize model parameter selection (Appendix B).

## 4. Discussion

The main advantage of PulseHeart is that the training required for the examiner is limited to the ability to record a short video of the transthoracic four-chamber view with sufficient quality. As proof of concept, the results obtained here demonstrate that this end-to-end approach is feasible and exhibits good performance. Such an EF classification algorithm, either AutoML or not, can be considered to aid in categorizing heart failure from the perspective of therapeutic guidance and prognostic.

Our aim was not to directly compare the metrics to those of the original Stanford study but to verify whether our proposed approach could exhibit acceptable performance. We chose to undersample the majority class to keep the computational cost within reasonable limits [14], which was necessary to carry out the present study. Before balancing, we selected an intermediate unbalanced dataset from the Stanford source that would be suitable for our proof of concept. We first discarded the tiny fraction (0.4%) of the source dataset for which the label was problematic in the a posteriori re-evaluation by five experts from Stanford [10]. We acknowledge that some of these 40 labels were probably correct, but others were judged to be patent label errors. Our intention was, therefore, not to remove difficult cases but rather to obtain a dataset with a high rate of correct labels. Many difficult cases remained in our intermediate dataset, and this was confirmed by our five-cardiologist re-evaluation. We also selected the videos with the same predominant size and frame rate, which corresponds to most real-world situations. Videos that were too short or too long were discarded because we wanted to evaluate an approach that included standardized recording without possibly biased human decisions to record more or fewer beats than usual.

For any attempt to compare PulseHeart to EchoNet-Dynamic, it would be necessary to use the full 10,030 video set for PulseHeart or to train the EchoNet-Dynamic model with our balanced datasets. This would also require k-fold cross-validation [15], which was not performed for EchoNet-Dynamic and is not available in Vertex AI for what concerns our study. Engaging in this comparison does not appear justifiable because EchoNet-Dynamic does not constitute a corresponding benchmark. It differs primarily in that it is not an end-to-end approach and, therefore, requires the presence of health personnel with significant expertise for the specific task of left ventricular cavity delineation when used for prediction in new patients. PulseHeart can, therefore, be an interesting solution in a certain number of clinical situations. Additionally, EchoNet-Dynamic only considered one type of classification, did not use balanced datasets, and was not tested to distinguish between the three classes of EF that are presently considered of clinical importance.

Some other limitations should be mentioned before interpreting the results in more detail. Limitations exist if one wishes to statistically compare the performance with the S, M, and L sets for the three classifications. Not only would this again involve k-fold cross-evaluation, but the study design was not suitable for this unplanned comparison. Consistent with our primary objective, the PulseHeart was tested for three classification types that could be considered in different clinical settings. The original design was to use the S-set for the rEF vs. nrEF binary classification, the L-set for the npEF vs. pEF binary classification, and the M-set for the ternary classification. We continued our investigation by training the models with the two other datasets for each type of classification, which led to some interesting observations. The same test dataset for the nine models would have been theoretically preferable for analyzing performance in function of the balancing method. The small and medium test datasets contain only videos from the large test dataset. After reduction via the random balancing process, these datasets, on average, continue to reflect their common origin. Therefore, we did not rerun the experiments with the large test dataset. This would have required additional time-consuming and expensive AutoML calculations without significantly improving our metric estimates or changing the validity of the scientific conclusion.

Another limitation is that in the AutoML approach, as well as in commercially available patented applications [16], there is no access to model details or parameter tuning. At the time of our study, Google Cloud Platform Vertex AI included, among the video autoML options, a classification module that was not available in Microsoft Azure or Amazon Web Services SageMaker. AutoML refers to any automated process of building ML models, consisting of searching for a suitable model, tuning hyperparameters, preparing data, and deploying the model. AutoML services are constantly developing, and applications are described in the non-medical and medical domains [17,18,19,20]. The main disadvantage of AutoML is the black-box effect. Only very general considerations and examples are provided for the Video Classification on Google Cloud. We know that Vertex AI uses MoViNets and that the pre-training was on the Kineticcs 600 video dataset, but we lack a precise description of how AutoML worked in our experiments, nor can we provide an analysis of the resulting models, including hyperparameter values and training loss curves. However, this opacity was not a real hindrance in the present study aimed at proving the concept and assessing the potential of the PulseHeart approach. AutoML accomplished the task quickly, efficiently, and at a reasonable cost. A further inconvenience of the black-box effect is that there is no way to understand why the completion times of the algorithms are not proportional to the training dataset size, with the M-set being the longest to train. For this, we need to know if, in all AutoML experiments, the model was the same, the hyperparameters were similar, and the training loss curves were equally satisfactory. In our independent PyTorch trial, where these conditions were met, the completion time was proportional to the size of the dataset and the size of the model. This strengthens the hypothesis of different-sized models to explain the observation in AutoML experiments.

Despite these limitations, this study demonstrates that the PulseHeart concept is feasible, with a performance level generally judged acceptable in the field of medical imaging, where imperfect labeling and a relatively low number of observations are frequently encountered. In general, the best-performing dataset was the smallest (1658 videos), balanced for the 829 rEF cases representing the minority. The largest dataset (3064 videos) is balanced for npEF and is the second best in terms of performance.

The global metrics used in this study deserve some comments. Accuracy is a well-recognized metric, but the F1 score is more representative in a dataset that is not fully balanced. The observed values of these two metrics do not differ much in the models trained here. Likewise, the PRC AUC score could be preferred to the ROC AUC score, which is the usual reference value. As expected, the two are deeply connected in our observations, but PR curves are deemed more informative when dealing with skewed datasets [21]. For the ROC curve graphs of the npEF vs. pEF classification, we considered npEF positive because, in medicine, a test result indicative of illness is reported as positive by convention.

If we look at the class metrics, we can draw more detailed conclusions. For the binary classification to identify rEF (S-R-NR model), the positive predictive value (precision 1) is 0.842, the sensitivity is 0.894, the F1 score is 0.867, and the specificity is 0.831. The high sensitivity is a satisfactory result. Indeed, rEF cases should be missed as little as possible in a general patient population, as they can be the warning sign of serious cardiac problems. The specificity is such that few nrEF patients would undergo further cardiac investigations. For the binary classification to identify npEF (L-NP-P model), the positive predictive value (precision 1) is 0.888, the sensitivity is 0.761, the F1-score is 0.817, and the specificity is 0.891.

We observed, in general, lower metric values for the ternary classification. This was essentially marked for the class metrics concerning mEF. In contrast, in the M-3-C model where dataset balance and classification match, only 1 out of 169 pEF videos was classified as rEF, and only 7 out of 169 rEF were classified as pEF.

A binary approach is often preferred in ML applications for medical diagnosis concerning disease stages. For algorithms without transfer learning in Alzheimer’s disease, a ternary classification was described using constructed cascaded convolutional neural networks [22], and several attempts of binary classification for disease vs. the normal status were reported, with, for instance, a model using a 3D VGG variant convolutional network [23]. For grading glioma, a modular deep-learning pipeline using an ensemble of convolutional neural networks was proposed, realizing two binary classifications in a cascade [24]. However, these authors state that the worst tumor stage (grade IV) is clearly distinct from grades III and II, which are less easy to separate. A variety of other deep-learning algorithms were tested for image multi-classification of brain tumors, as reviewed in an article where the authors proposed their own models [25]. In the present study, the attempts of ternary classification might be hampered by the skewed smooth EF distribution where multimodality does not appear clearly (Figure 3). Our clinical re-evaluation also points to the difficulties of isolating a gray zone between frankly pathological states and normality, even in the presence of reliable labels.

We mentioned that the side observations obtained in the six models where the way of balancing does not match the type of classification must be interpreted with caution. Nevertheless, we cannot neglect that, as shown in Table 3 and Table 4, improved metrics are sometimes observed in these mismatches. Figure 1c shows that the majority of npEF cases are in the S-NP-P and M-NP-P models. They have a higher sensitivity for identifying npEF than the L-NP-P model balanced by undersampling pEF. However, their lower specificity implies that many patients with pEF would undergo more extensive cardiac investigations. Only statistically robust comparisons, ideally based on k-fold cross-validation, are suitable to determine a dataset balancing strategy adapted to a given clinical situation. These should be included in future attempts to improve the models through a non-AutoML approach.

The power of transfer learning in medical imaging based on a pre-training of colored images is illustrated by the performances reported in a wide spectrum of medical conditions using grayscale images produced by techniques such as computed tomography, mammography, magnetic resonance imaging, or chest X-rays [26,27,28]. Similarly, in the present study, the model was pre-trained on color video sets and applied with success to grayscaled clips.

We can wonder why this algorithm works without providing any information about the left ventricular cavity or the heart’s cyclic evolution. The first point is to note that the four-chamber view is, by convention, pre-framed by the performer of the examination and oriented such that the left ventricle occupies approximately the same position on each video. The almost echo-free zone generated by the presence of ultrasound-permeable blood in the cavity can thus be tracked by the algorithm. This is only a conjecture because here, we lack a visual explanation by means of the saliency methods that, in static medical images, allow localizing the region of interest of the automated search process on a heatmap [29,30,31,32]. It is also possible that other cyclic phenomena that affect the cardiac structures contribute to the confidence score, such as the left ventricular wall contraction, the complex relationship between the systolic and diastolic function, the left atrial filling and emptying, or the degree of interaction between the right and left ventricle. Another point is that the principle of EF calculation from a four-chamber echocardiographic view is based on a three-dimensional model extrapolated from a single plane. Ultrasound images are constructed from the reception of echoes coming from a slice perpendicular to the surface of the transducer and determined by its orientation. These echoes, particularly the backscattered ones, carry clues about the part of the heart adjacent to the reconstructed slice. This information can be correlated with the patient’s EF. Furthermore, the power of transfer learning to classify cyclic events on video is well demonstrated in action recognition tasks. Therefore, the PulseHeart approach, which uses a “scene recognition task”, can take advantage of the repetition of the cardiac cycle in the video sequences.

For future research, models based on the PulseHeart approach can be developed using SDKs such as TensorFlow or PyTorch. This can be done locally or on a cloud ML platform. Unreasonable costs encountered in commercial platforms could be avoided, especially if k-fold cross-validation is required and extensive experimentation is needed to refine the models. As we demonstrate in Appendix B, training loss curves can then be observed, hyperparameters tuned, and different kernels tested. Overfit can be detected more easily by introducing a validation phase into the training process. We can consider combining other data modalities (such as electrocardiogram, NT-proBNP, chest X-ray, or MRI) to enhance the performance of the classifier. Testing the predictions on external validation datasets from different centers may be considered to assess the generalization ability of this approach. One conceivable development is to articulate classical ML SDKs with quantum computing SDKs such as Pennylane and Qiskit to build a transfer learning classical-quantum hybrid video classifier. For example, the “extended” circuit version of MoViNet that we built on PyTorch allows the insertion of a doctored parameterized quantum circuit. The complexity of the videos and the possibility of interactions between the changing cardiac structures during the cardiac cycle are reasons to expect increased trainability and/or performance in such hybrid models [33,34,35]. These were already tested for static grayscale images in the medical field [36,37,38,39]. Transfer learning video classification of EF may be considered for other procedures, like transesophageal echocardiography, contrast ventriculography, magnetic resonance imaging, and computed tomography.

## Figures and Tables

**Table 1:** Balanced datasets.

Dataset	Label *	Support (Train)	Support (Test)
S-set: 1658 videos Balanced for rEF vs. nrEF	rEF	621	208
	mEF	197	64
	pEF	425	143
M-set: 2109 videos Balanced for all three classes	rEF	534	169
	mEF	534	169
	pEF	534	169
L-set: 3064 videos Balanced for npEF vs. pEF	rEF	621	208
	mEF	534	169
	pEF	1155	377

* Initial human label based on EF estimated by Simpson method.

**Table 2:** Model registry of video classification by AutoML, with job duration.

Model	Dataset	Classification	Job Duration
S-R-NR	S-set	Binary: rEF vs. nrEF	3 h 49 min
S-NP-P	S-set	Binary: npEF vs. pEF	3 h 18 min
S-3-C	S-set	Ternary: rEF, mEF, pEF	3 h 53 min
M-R-NR	M-set	Binary: rEF vs. nrEF	6 h 9 min
M-NP-P	M-set	Binary: npEF vs. pEF	5 h 9 min
M-3-C	M-set	Ternary: rEF, mEF, pEF	4 h 29 min
L-R-NR	L-set	Binary: rEF vs. nrEF	2 h 50 min
L-NP-P	L-set	Binary: npEF vs. pEF	2 h 34 min
L-3-C	L-set	Ternary: rEF, mEF, pEF	2 h 49 min

**Table 3:** Metrics observed in binary video classification.

Classifier	Metrics	S-Set	M-Set	L-Set
Binary: rEF vs. nrEF	All labels:			
	Accuracy	0.863	0.822	0.845
	Balanced accuracy	0.863	0.796	0.816
	ROC AUC	0.939	0.896	0.904
	PRC AUC *	0.935	0.904	0.926
	Label: rEF			
	Precision	0.842	0.745	0.708
	Recall (sensitivity)	0.894	0.710	0.736
	F1 score	0.867	0.727	0.722
	Label: nrEF			
	Precision	0.887	0.858	0.891
	Recall (specificity)	0.831	0.879	0.896
	F1 score	0.858	0.868	0.893
Binary: npEF vs. pEF	All labels:			
	Accuracy	0.896	0.854	0.829
	Balanced accuracy	0.870	0.829	0.826
	ROC AUC	0.952	0.919	0.917
	PRC AUC *	0.948	0.924	0.918
	Label: pEF			
	Precision	0.924	0.808	0.792
	Recall (specificity)	0.769	0.746	0.891
	F1 score	0.840	0.775	0.839
	Label: npEF			
	Precision	0.883	0.875	0.888
	Recall (sensitivity)	0.971	0.911	0.761
	F1 score	0.925	0.893	0.817

* Appears under the name “average precision” in Vertex AI output.

**Table 4:** Metrics observed in ternary video classification.

Classifier	Metrics	S-Set	M-Set	L-Set
Ternary: rEF, mEF, pEF	All labels:			
	Accuracy	0.759	0.700	0.745
	Balanced accuracy	0.740	0.829	0.817
	PRC AUC *	0.856	0.795	0.829
	Label: rEF			
	Precision	0.883	0.749	0.697
	Recall	0.837	0.811	0.851
	F1 score	0.859	0.778	0.766
	Label: mEF			
	Precision	0.295	0.545	0.403
	Recall	0.438	0.716	0.651
	F1 score	0.352	0.619	0.498
	Label: pEF			
	Precision	0.699	0.618	0.778
	Recall	0.944	0.959	0.950
	F1 score	0.804	0.752	0.855

* Appears under the name “average precision” in Vertex AI output.

**Figure A1:**
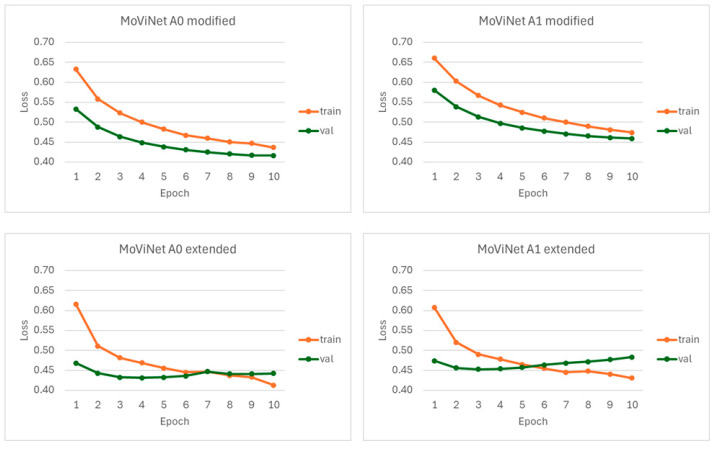
Loss curves observed for the rEF vs. nrEF classifier. The MoViNet models A0 and A1 were each tested either with a modification involving the last Conv3d layer or by adding a linear layer to the backbone.

**Figure A2:**
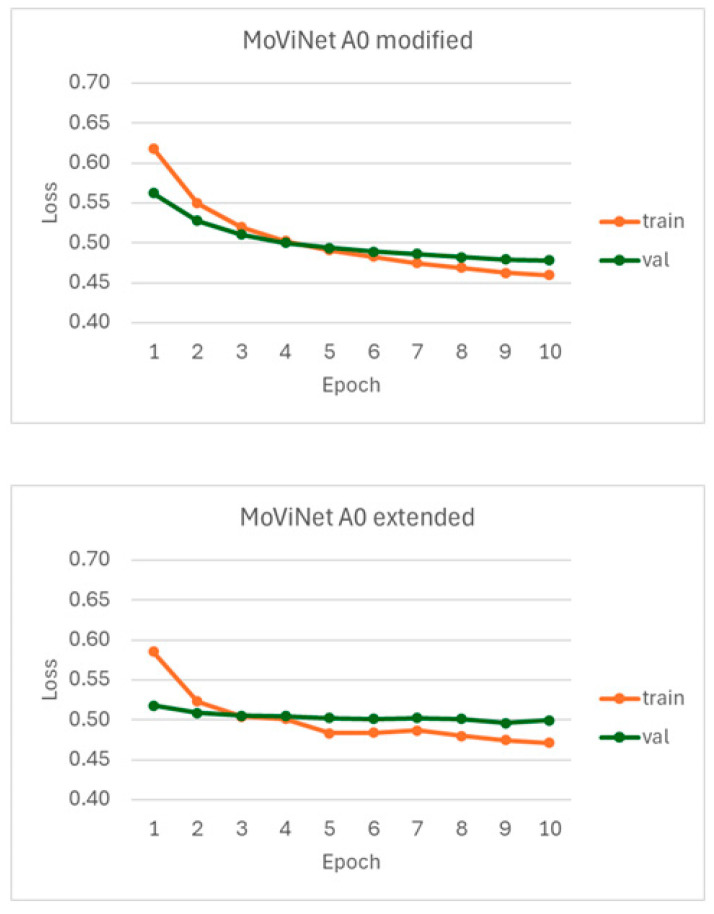
Loss curves observed for the npEF vs. pEF classifier using the MoViNet model A0 after backbone modification or extension.

**Figure A3:**
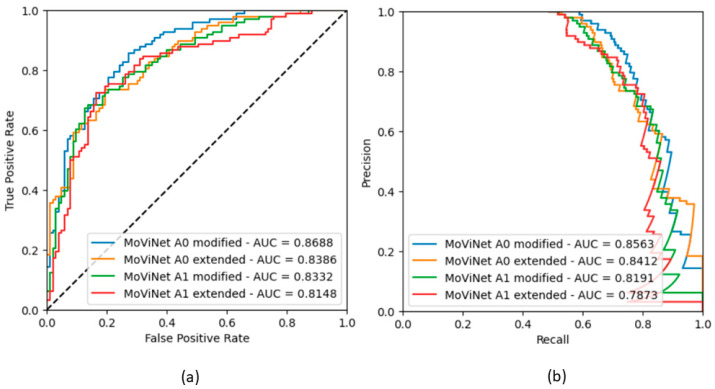
Binary classifier rEF vs. nrEF: (**a**) ROC curves obtained in the four models; (**b**) Corresponding precision-recall curves.

**Figure A4:**
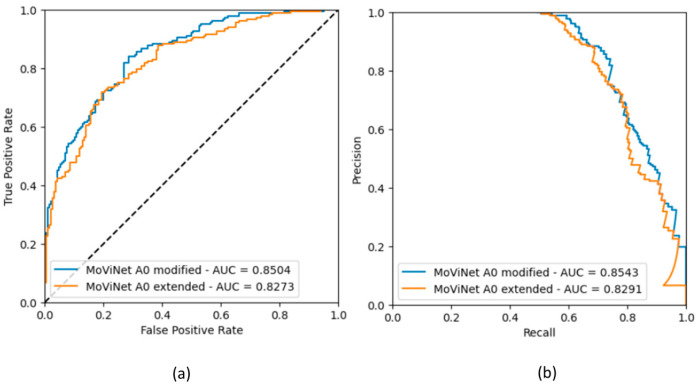
Binary classifier npEF vs. pEF: (**a**) ROC curves obtained in the two models; (**b**) Corresponding precision-recall curves.

## Data Availability

Files containing the managed dataset, the annotation sets, and the detailed re-evaluation ratings are available online: https://github.com/pulseheart/PulseHeart-AutoML (accessed on 12 October 2023). The Pytorch implementation with Python notebooks under Apache License 2.0 can be found at https://github.com/pulseheart/PulseHeart-PyTorch (accessed on 4 April 2024)). The videos can be downloaded from the Stanford AIMI Shared Dataset https://echonet.github.io/dynamic/index.html (accessed on 12 October 2023) after login and agreeing to the Stanford University Dataset Research Use Agreement. A description of the video clip classification models can be found on GitHub at the official site of MoViNet https://github.com/tensorflow/models/blob/master/official/projects/movinet/README.md (accessed on 12 December 2013).

## Appendix A

**Table A1:** Clinical Re-evaluation.

AVI File	EF	Pred	Rater 1	Rater 2	Rater 3	Rater 4	Rater 5	Panel
0X7AB682A3B8DEC28A	22.8	pEF	rEF	rEF	rEF	rEF	rEF	rEF
0X9756BC052F770E5	24.8	nrEF	mEF	rEF	rEF	rEF	mEF Q	rEF
0X73E9825196EB26F9	27.2	pEF	rEF A	rEF Q A	rEF A	mEF Q A	pEF	rEF
0X5216E8EAC6638EC5	27.7	nrEF	rEF	rEF	rEF	rEF	rEF	rEF
0X5BFC2EC0D445EA65	28.1	nrEF	rEF	mEF	rEF Q A	rEF A	pEF Q	rEF
0X2FAFDA5737784951	28.4	nrEF	rEF	rEF	rEF	rEF	rEF	rEF
0X70C7E7E952C28C1	28.8	nrEF	mEF	mEF	mEF	pEF	mEF Q	mEF
0X2F178A2E89C73B5	29.2	pEF	pEF	pEF Q	mEF Q	mEF Q	mEF Q	mEF
0X2B5619B4EDE8F1B8	29.3	pEF	mEF	mEF	mEF	mEF	mEF	mEF
0X260767549892A590	30.4	nrEF	rEF Q A	mEF Q	rEF Q	mEF Q	rEF Q	rEF
0X1FC4816F238B726E	30.6	pEF	rEF	rEF	rEF	rEF Q	mEF	rEF
0X28C73B18FDF845BC	30.9	pEF	pEF A	mEF	rEF Q	rEF A	mEF Q A	mEF
0X32312BC4DF1CD8A3	31.1	nrEF	mEF	rEF	rEF	mEF	rEF	rEF
0X3503A92D7637451	31.8	nrEF	rEF Q A	rEF Q	rEF	rEF	rEF Q	rEF
0X2007E059C9C83B68	32.6	pEF	pEF Q	pEF Q	rEF	mEF Q	mEF	mEF
0X10AD385C206C85C	32.8	pEF	rEF Q	mEF Q	rEF	mEF Q	mEF Q	mEF
0X7329DF92A352EC62	33.2	nrEF	pEF	mEF	mEF	rEF	rEF	mEF
0X4A49AE73D48ED549	34.9	nrEF	rEF Q	mEF Q	mEF	rEF Q	mEF Q A	mEF
0X302995669B66A122	34.9	nrEF	pEF Q	mEF Q	rEF Q	rEF Q	rEF	rEF
0X7EEA66DBE251854B	35.2	nrEF	rEF A	rEF A	rEF A	mEF A	rEF	rEF
0X575A1E4C8C441849	36.1	nrEF	rEF	mEF Q	mEF Q	rEF Q	rEF	rEF
0X560AC3ED5C9AA949	36.3	pEF	rEF	rEF	rEF Q	rEF	rEF	rEF
0X5C0BCAA2FB4FF1B4	37.4	pEF	rEF A	mEF A	mEF Q A	mEF Q	mEF Q A	mEF
0X6050E603BC35F0D	37.7	nrEF	rEF	rEF Q A	rEF	pEF	pEF Q A	rEF
0X3285FE374F563092	37.8	nrEF	mEF	mEF	mEF	mEF	rEF	mEF
0X30AFF793AC29BD2B	37.9	nrEF	rEF	rEF	rEF	rEF	mEF	rEF
0X5C7B3A4A12245C5E	38.0	nrEF	pEF Q	mEF Q	mEF Q	mEF Q	rEF Q	mEF
0X5D78417E9211CC18	38.1	nrEF	mEF Q	mEF Q	rEF Q	mEF	mEF Q A	mEF
0X5042C6AB36212224	38.4	nrEF	rEF	mEF	rEF Q	mEF	mEF Q	mEF
0X4D17A70DB464D7EB	39.2	pEF	mEF A	mEF Q A	rEF	rEF	mEF	mEF
0X5A887EDA76C326E9	39.2	nrEF	rEF Q A	rEF	rEF	rEF	rEF	rEF
0X7BA9FD251A48D45B	39.3	nrEF	rEF	rEF	rEF	rEF	rEF	rEF
0X31E539C27D120BDE	39.9	nrEF	mEF	mEF	mEF Q	rEF	mEF Q	mEF
0X11C89001BEF939E2	40.4	pEF	rEF Q	rEF	rEF	rEF	rEF	rEF
0X44F9A80B05DFC224	41.2	rEF	mEF QA	pEF	rEF Q	rEF Q	pEF Q	mEF
0X775319C257A48042	41.4	pEF	mEF Q	mEF Q	rEF Q	rEF Q	rEF	rEF
0XBD39E52A48060D2	42.1	pEF	rEF	mEF	rEF	rEF	rEF	rEF
0X3C63C23E5B0823D	43.2	pEF	rEF Q	mEF Q	rEF Q	rEF Q	rEF	rEF
0X38638F441D35402	43.3	rEF	pEF	mEF	mEF	rEF	rEF	mEF
0X4D383DD98BD6CD12	43.4	pEF	mEF	mEF	rEF	rEF	rEF	rEF
0X4704159CFC29D4C3	43.9	pEF	rEF A	mEF A	rEF	rEF A	mEF Q A	rEF
0X2C871D22AD5EAD1A	44.3	rEF	pEF	mEF	mEF	pEF Q	rEF	mEF
0X69B8DBAA13F1442B	44.6	rEF	mEF	mEF Q	rEF	mEF Q	pEF	mEF
0X1337E8945A141439	44.7	pEF	mEF	mEF	rEF	rEF	rEF	rEF
0X13F7CAB4C719ACA3	45.0	rEF	pEF	pEF	pEF	mEF	pEF	pEF
0X114B58E6B34E55F1	45.1	rEF	pEF	pEF Q	pEF Q	rEF Q	mEF Q	pEF
0X71FE0206D64EDD39	45.3	rEF	pEF	pEF Q	pEF	pEF Q	pEF	pEF
0X7540B06840A33DBF	45.4	rEF	pEF	pEF A	pEF	pEF	mEF	pEF
0X75BA1623CCCF0652	45.4	pEF	mEF	mEF	rEF Q	rEF Q	rEF Q	rEF
0X1C1A328EA29B6CC3	45.6	rEF	pEF Q	pEF	mEF Q	pEF Q	pEF	pEF
0X1F30E6AC3FE50EE3	46.0	rEF	pEF Q	pEF Q	rEF Q	mEF Q	mEF Q	mEF
0X6CA712DE9D936CB3	46.4	rEF	pEF A	pEF A	pEF	pEF Q	pEF	pEF
0XD537CD5A04B8C43	46.7	rEF	pEF	pEF	pEF Q	mEF	mEF	pEF
0X25D970C75A57B3F2	46.7	rEF	pEF	pEF Q	pEF Q	pEF Q	pEF	pEF
0X3077040EC90D916D	46.8	rEF	mEF	mEF	mEF Q	mEF	mEF Q	mEF
0X40551ED55932933D	46.9	rEF	pEF	mEF Q	mEF Q	mEF Q	mEF	mEF
0X15E8BE2AE8C05C88	46.9	rEF	pEF	pEF	pEF	rEF Q	pEF	pEF
0X6B0A6A101C2DA474	47.0	rEF	pEF	pEF	mEF	mEF	mEF	mEF
0X57A074E488CFB7AC	47.2	rEF	pEF Q	pEF Q	rEF	pEF	pEF	pEF
0X3902FE711F8B581B	48.0	rEF	pEF	pEF	mEF	pEF	mEF Q	pEF
0X35607DFD91E00F2B	48.0	rEF	rEF A	mEF	mEF	mEF	rEF	mEF
0X65ACA3F8B770AAD7	48.1	rEF	pEF	pEF	rEF Q	mEF Q	pEF Q A	pEF
0X2AFCAC3003694C4D	48.1	pEF	mEF Q	rEF Q	rEF Q	mEF Q	rEF Q	rEF
0X6A11E31F14ADFDEE	48.3	rEF	pEF	mEF	rEF Q	mEF	mEF	mEF
0X63EFA5F7FFFB0014	48.4	rEF	mEF	mEF	pEF	mEF	mEF	mEF
0X5440A5C9A8CACA49	48.9	rEF	pEF QA	pEF Q	pEF Q A	pEF Q A	pEF Q	pEF
0X1B4F427BC662B727	48.9	pEF	pEF Q	mEF	rEF Q	rEF Q	rEF	rEF
0X7923B6B4614AF456	49.1	rEF	rEF	mEF	mEF Q	mEF Q	mEF Q	mEF
0X73CBCADA2191104C	49.5	rEF pEF	pEF	pEF	mEF Q	rEF Q	pEF	pEF
0X26B209C0083A4AD5	49.8	rEF	pEF	pEF	mEF Q	mEF Q	mEF	mEF
0X3B80677CE0873E50	49.8	rEF	pEF	pEF	pEF	pEF	mEF	pEF
0X4191ACD0157311E5	50.1	npEF	mEF	rEF	rEF Q	mEF Q	mEF Q	mEF
0X16AF26F9A372EEDE	50.5	npEF	mEF	mEF	mEF	pEF	pEF	mEF
0X2962BA442DE9E45B	51.5	npEF	pEF	pEF	pEF	mEF	pEF	pEF
0X5AC82E1FBDF09C04	51.9	npEF	pEF	pEF	rEF	mEF Q	mEF	mEF
0X7871A13A25E72847	52.5	npEF	pEF	pEF	mEF Q	mEF Q	mEF Q	mEF
0X40253981E97848E5	52.9	npEF	mEF	pEF	mEF	mEF Q	mEF	mEF
0X731BBCC68C30384D	53.4	npEF	pEF	pEF	pEF Q	mEF	pEF	pEF
0X3A3085150FD2D6E8	53.5	npEF	pEF	pEF	pEF Q	rEF	pEF	pEF
0X113195610E41EF2	54.7	npEF	mEF Q	mEF	pEF	mEF	mEF	mEF
0X5DDE9E68BB099303	55.1	npEF	pEF QA	pEF	pEF Q	mEF Q	pEF Q	pEF
0X210265FBDA5360AE	55.1	npEF	pEF	pEF	pEF	pEF	pEF	pEF
0X3BFFB8615C86AE75	55.1	npEF	pEF	mEF Q	rEF Q	rEF	rEF	rEF
0X6E1F0B0B5831B801	55.7	rEF npEF	pEF	pEF	pEF	mEF Q	pEF Q	pEF
0X12807854DFA9CC01	56.8	npEF	pEF	pEF	rEF Q	rEF Q	pEF	pEF
0X5843363A84693349	57.0	npEF	pEF Q	pEF	mEF Q	pEF Q	mEF	pEF
0X77B0F03C4F1E0315	57.2	npEF	pEF	pEF	pEF	pEF Q	mEF Q	pEF
0X41ECEC7AAEEFD0E6	57.3	rEF	pEF Q	pEF Q	pEF Q	pEF Q	pEF Q	pEF
0X6AB214EB6B92DC02	57.3	npEF	pEF	pEF	pEF	mEF	pEF	pEF
0X2489A40319D6990E	57.8	rEF npEF	pEF Q	pEF	pEF	pEF	pEF	pEF
0X2841EE2AE1958F10	58.2	npEF	pEF	pEF	pEF	pEF	pEF	pEF
0X445575CFEECB0986	58.7	npEF	pEF	pEF	pEF	mEF Q	pEF	pEF
0X166B717BBC2ECADA	59.0	npEF	pEF Q	pEF	pEF Q	pEF	pEF	pEF
0X7BF746EB936C65BE	59.1	npEF	pEF Q A	pEF Q A	pEF Q	pEF A	pEF	pEF
0X7A77DF8AACD6E023	59.2	npEF	pEF	pEF	pEF	pEF Q	pEF	pEF
0X8E2FCF5187C4872	61.1	npEF	pEF	pEF	pEF	pEF	pEF	pEF
0X1039B49145DF4F25	62.4	rEF npEF	pEF	pEF	pEF	mEF Q	pEF	pEF
0X5FBBC76F7AD9FB4D	62.6	npEF	pEF A	pEF A	pEF	pEF A	mEF Q A	pEF
0X2ECE3ECC0BF62256	63.1	npEF	pEF Q	pEF Q	mEF Q	pEF	pEF	pEF
0X6A672DABBE9F8660	63.3	npEF	pEF	pEF Q	pEF Q	pEF	pEF	pEF
0X343CEAA877051407	63.5	npEF	pEF	pEF	pEF	pEF Q	pEF	pEF
0X73F6DA33A9F3A272	64.1	npEF	pEF	pEF	pEF Q	pEF	pEF	pEF
0X127D3AEEA73EDE76	64.7	npEF	pEF	pEF	pEF	pEF	pEF	pEF
0X5B9C0EEB93E0BE10	65.3	npEF	pEF	pEF	pEF	pEF	pEF	pEF
0X3E56DED8582F762B	65.7	npEF	pEF	pEF	pEF	pEF Q	pEF	pEF
0X17828CD670289D36	66.9	npEF	pEF Q	pEF Q	pEF	pEF	pEF	pEF
0X4D2FF488DD4EC6BD	70.0	npEF	rEF	pEF Q	pEF Q	pEF Q	pEF Q	pEF

EF: left ventricular ejection fraction obtained by the Simpson method. Pred: EF class predicted by classifier(s). Raters: Q indicates that the rater reported poor technical quality; A indicates that the rater reported arrhythmia. Panel: five-rater judgement.

## Appendix B

An implementation based on software development kits (SDKs) programmed in Python was carried out.

### Appendix B.1. Dataset and Data Curation

We used the balanced S-set and L-set described above to test binary classifiers, respectively, of rEF v. nrEF and npEF vs. pEF. Train/validation/test split was chosen instead of train/test split. The original split labels of the Stanford dataset were retained. The models we considered for experimentation did not accept an incomplete last batch in the training and validation phases. The final data subsets obtained according to this request are detailed in Table A2. With the models having been pre-trained on clips of 50 frames, the study AVI videos were reduced to 50-frame MP4 sequences by modifying the frame rates. We used ffmpeg-python for this purpose [40].

**Table A2:** Balanced datasets used in the open-access software study.

Dataset	Support (Train)	Support (Validation)	Support (Test)
1641 videos balanced for rEF vs. nrEF	619 621	104 96	98 103
3040 videos balanced for npEF vs. pEF	1148 1152	192 188	177 183

In the process of vectorization, the video files were resized to (172, 172), grayscaled, converted to float 32 values, and normalized using mean = [0.43216, 0.394666, 0.37645] and standard deviation = [0.22803, 0.22145, 0.216989].

### Appendix B.2. Training

After a series of preliminary experiments, the pre-trained models for video classification offered on the official PyTorch SDK [41] were not further explored due to their unsatisfactory performance. The MoViNet-pytorch GitHub directory [42] allowed us to access models pre-trained on the Kinetic 600 dataset. Among them, MoViNet A0, in its basic version, could be trained in a reasonable time and without working memory issues. The larger MoViNet A1 basis model could also be trained on the small dataset. The corresponding stream versions had to be abandoned due to a lack of convergence. For each model, we obtained a final binary classification by two approaches, “modified” and “extended”. In the first, the output dimension of the last Conv3d layer was set to 2 instead of 600. The parameters of this layer were used by the optimizer in the training process. In the second approach, the last layer of the backbone was left intact, its parameters remaining frozen. A final linear layer (600, 2) was added, whose parameters were used by the optimizer.

All models were trained on CPU using 42 as random seed, cross-entropy loss as cost function, and Adam [43] as optimizer. Based on the exploratory results, the hyperparameters for an equal comparison of the models were set as follows: learning rate = 0.01, batch size = 20, and number of epochs = 10. Each train epoch was followed by a validation epoch. The final weights for a prediction model were those corresponding to the first occurrence of the minimum loss observed during the validation phases.

The loss curves concerning the rEF vs. nrEF classifier are shown in Figure A1. The execution times for models A0 modified, A0 extended, A1 modified, and A1 extended were 273 min, 265 min, 507 min, and 508 min, respectively.

The loss curves concerning the npEF vs. pEF classifier are shown in Figure A2. The execution times for models A0 modified and A0 extended were 580 min and 525 min, respectively.

### Appendix B.3. Metrics

The metric values are presented in Table A3. For each classifier, the highest accuracy, ROC AUC scores, and PR AUC scores are observed with the model MoViNet A0 modified. Figure A3 and Figure A4 show the ROC and PR curves.

**Table A3:** Metrics observed in binary video classification for the test dataset.

Metrics	MoViNet A0 (Modified) rEF vs. nrEF	MoViNet A0 (Extended) rEF vs. nrEF	MoViNet A1 (Modified) rEF vs. nrEF	MoViNet A1 (Extended) rEF vs. nrEF	MoViNet A0 (Modified) npEF vs. pEF	MoViNet A0 (Extended) npEF vs. pEF
All labels:						
Accuracy	0.776	0.756	0.761	0.771	0.750	0.728
Balanced accuracy	0.777	0.756	0.761	0.772	0.750	0.738
ROC AUC	0.869	0.839	0.833	0.815	0.850	0.827
PRC AUC	0.856	0.841	0.819	0.787	0.854	0.829
Label: rEF						
Precision	0.785	0.758	0.745	0.783	0.737	0.686
Recall (sensitivity)	0.745	0.735	0.776	0.735	0.769	0.830
F1 score	0.764	0.746	0.760	0.758	0.753	0.751
Label: nrEF						
Precision	0.769	0.755	0.778	0.761	0.764	0.791
Recall (specificity)	0.806	0.777	0.748	0.806	0.731	0.629
F1 score	0.787	0.766	0.762	0.783	0.747	0.701
